# Multifractal Properties of Time Series of Synthetic Earthquakes Obtained from a Spring-Block Model

**DOI:** 10.3390/e25050773

**Published:** 2023-05-09

**Authors:** Ana M. Aguilar-Molina, Alejandro Muñoz-Diosdado, Alfredo Salinas Martínez, Fernando Angulo-Brown

**Affiliations:** 1 Unidad Profesional Interdisciplinaria de Biotecnología, Instituto Politécnico Nacional, Mexico City 07340, Mexico; 2 Departamento de Física, Escuela Superior de Física y Matemáticas, Instituto Politécnico Nacional, UP Zacatenco, Mexico City 07738, Mexico

**Keywords:** self-organized criticality, spring-block, seismicity

## Abstract

With the spring-block model proposed by Olami, Feder, and Christensen (OFC), we obtained a time series of synthetic earthquakes with different values of the conservation level (β), which measures the fraction of the energy that a relaxing block passes to its neighbors. The time series have multifractal characteristics, and we analyzed them with the Chhabra and Jensen method. We calculated the width, symmetry, and curvature parameters for each spectrum. As the value of conservation level increases, the spectra widen, the symmetric parameter increases, and the curvature around the maximum of the spectra decreases. In a long series of synthetic seismicity, we located earthquakes of the greatest magnitude and built overlapping windows before and after them. For the time series in each window, we performed multifractal analysis to obtain multifractal spectra. We also calculated the width, symmetry, and curvature around the maximum of the multifractal spectrum. We followed the evolution of these parameters before and after large earthquakes. We found that the multifractal spectra had greater widths, were less skewed to the left, and were very pointed around the maximum before rather than after large earthquakes. We studied and calculated the same parameters and found the same results in the analysis of the Southern California seismicity catalog. This suggests that there seems to be a process of preparation for a great earthquake and that its dynamics are different from the one that occurs after this mainshock based on the behavior of the parameters mentioned before.

## 1. Introduction

Earth’s seismicity is one of the most representative examples of systems that exhibit self-organized criticality (SOC) [1,2,3,4,5,6,7,8]. Seismic faults form fractal patterns [1,2,3,6,9,10]. From geological evidence, it has been shown that during an earthquake, the Earth’s crust breaks into fragments on a scale ranging from millimeters to hundreds of kilometers, having a fractal-like structure. Scale invariance is equivalent to a fractal distribution, which requires a power law dependence between the number of objects of a specific size with the size [10,11,12].

Although an explanation of these phenomena is given, the knowledge we possess does not allow us to predict earthquakes since the properties that govern them are not precisely known. It is necessary to improve the models that mimic the dynamics of plate tectonics to explain them under different configurations of tectonic compositions.

A possible interpretation of the Gutenberg-Richter law [13] is that the energy released during the earthquake grows exponentially with the size of the earthquake, which can be regarded as a manifestation of the self-organized critical behavior of the Earth’s dynamics. This law states that the rate of occurrence of earthquakes of magnitude M greater than m is given by the relation [14].

(1) $$\log_{10}\dot{N}\left(M>m\right)=a-bm,$$

where *a* and b are the GR parameters, $\dot{N}$ is the number of earthquakes per year with a magnitude M larger than m. The a-value measures the regional level of seismicity. The b-value is the slope of the straight line represented by Equation (1).

Bak, Tang, and Weisenfeld [1] introduced the concept of self-organized criticality to describe the global behavior of some complex systems. They understand that, by being self-organized, the system naturally evolves to a state without detailed specification of the initial conditions [1,2]. They explain that tectonic plate motion, providing the energy for earthquakes, would correspond to tilting sand grains in their model, which is similar to the sandpile model [6].

Olami, Feder, and Christensen (OFC) [15] proposed a non-conservative SOC model which maps directly to a two-dimensional version of the Burridge-Knopoff [16] spring-block model for earthquakes. This model displays robust SOC behavior over an extensive range of conservation levels, and the level of conservation impacts the power laws obtained. The dependence of the power laws on the level of conservation allows them to explain the wide variances in the Gutenberg-Richter law due to the variations of the elastic parameters [9,13,14]. OFC and other authors have shown that the values of the elastic parameter β around 0.2 are the values that better describe what happens in a real seismic subduction fault [9,13,14].

In this work, different studies were carried out with the synthetic seismicity series obtained from the OFC model. We worked with magnitude time series of $1\times 10^{7}$ synthetic earthquakes obtained with the algorithm of the OFC model with a wide range of conservation levels. We aimed to find properties of these time series associated with real seismicity [9,11,14]. We noted that the time series of synthetic earthquakes qualitatively reproduces the Gutenberg-Richter law and the stair-shaped plots for the cumulative seismicity graphics. The importance of qualitatively reproducing properties of real seismicity allows for studying synthetic catalogs without the problems that arise in real seismicity because synthetic catalogs are complete [9,14].

The synthetic time series have multifractal characteristics, and we analyzed them with the Chhabra and Jensen method (CHJ) [17] to obtain multifractal spectra. The multifractal spectra are skewed to the left, which indicates that these series are highly singular. We also obtained width, symmetry, and curvature parameters. The spectra widen, the values of the symmetry parameter increase, and the curvature values around the maximum of the spectra decrease as the values of the conservation levels increase.

We generated magnitude time series of $1\times 10^{7}$ data for a 0.2 value of the conservation level, and we located all of the large earthquakes with a magnitude equal to or greater than 8 and built windows around each earthquake. We calculated the multifractal spectra of each window after and before the large earthquake. We observed that the multifractal spectra have larger widths before than after the large earthquakes. The symmetry parameters show that the multifractal spectra of all windows are skewed to the left. We noticed that the multifractal spectra of the windows before the earthquake were more skewed to the left than the multifractal spectra after the earthquake, and the curvature parameter around the maximum of the multifractal spectra was larger before than after the earthquake.

In a similar way, we studied real seismicity with a time series of the Southern California seismicity catalog and located the earthquakes of magnitude greater than or equal to 7, and we also analyzed the seismicity before and after large-magnitude earthquakes.

The paper is organized as follows. In Section 2, we present a brief resume of the OFC spring-block model and multifractal theory; we briefly describe the CHJ method and the symmetry and curvature parameters. In Section 3, we present our results, and a discussion in Section 4. Finally, we present our conclusions.

## 2. Materials and Methods

### 2.1. The Olami, Feder, and Christensen Model

The OFC model is a non-conservative continuous model of two-dimensional self-organized criticality [15]. It consists of a system of four nearest neighbor blocks and two plates (upper-lower) interconnected by Hooke springs. Movement between the two plates displaces the blocks. The cellular automaton model starts when a block slides with a force greater than the maximum static friction. These forces will be redistributed to their nearest neighbors due to the movement of the block, and a chain reaction can be developed. The energy is transferred to the four nearest neighbors and then back to zero [15,18]. We refer the reader to references [15,18] for more details.

The most important parameter of the OFC model is the *β* conservation level, which measures the fraction of the energy that a relaxing block passes to its neighbors; for example, if β=0.2 each of the four neighbors is passed 0.2 of the energy available, that means that a fraction of 0.2 of the energy is lost, so the model is non-conservative.

### 2.2. CHJ Method

It is a direct calculation of the multifractal spectrum based on the formalism of the self-similar scaling properties [17]. The time series is normalized and is covered with boxes of equal length $\left(2^{n}\right)$ and we calculated the probabilities in each one of the boxes. Then the uni-parametric family of normalized measures is constructed as a function of the probabilities of the boxes and the q parameter Equation (2) [17]

(2) $${µ}_{i}=\frac{{\left[P_{i}(L)\right]}^{q}}{{\sum_{i}\left[P_{i}(L)\right]}^{q}}.$$

This *q*-parameter gives us information about the time series characteristics. For q>1, the most complex regions are enhanced, and for q<1, the same for the less complex regions, and the values for q=1 give us the fractal dimension. The fractal dimension f(α) Equation (3) and the Hölder exponent α Equation (4) is calculated from the uni-parametric family of the probabilities as a function of *q*. Then, for each *q*-value, both α and f(α(q)) are evaluated by cells of decreasing length. A detailed explanation of this model is given in references [17,19].

(3) $$f\left(\propto (q)\right)=\underset{L\to 0}{\lim}\frac{\sum {µ}_{i}(q,L)log[{µ}_{i}(q,L)]}{logL},$$

and the Hölder exponent is

(4) $$\alpha \left(q\right)=\underset{L\to 0}{\lim}\frac{\sum {µ}_{i}(q,L)log[P_{i}(q,L)]}{logL}.$$

### 2.3. Symmetry Parameter

**Figure 1:**
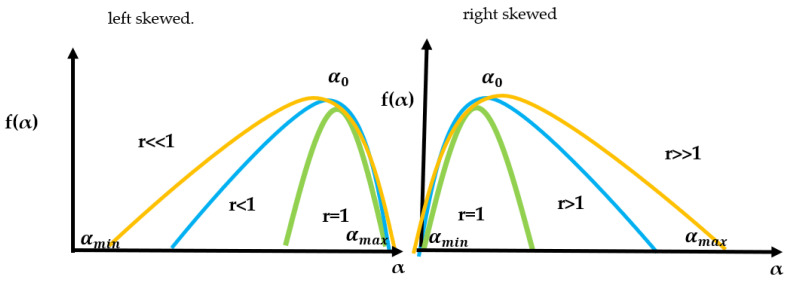
The different possible values of the symmetry parameter case and the location of $\alpha_{0}$, $\alpha_{min}$ and $\alpha_{max}$ parameters for the case r<<1 and r>>1. The location for the other cases is found in an analog form.

The symmetry parameter r gives us information on the asymmetry for the CHJ multifractal spectrum, defined as the absolute value of $\alpha_{max}-\alpha_{0}$ divided by $\alpha_{0}-\alpha_{min}$, $\alpha_{0}$ being the value where the spectrum reaches its maximum and $\alpha_{min}$, $\alpha_{max}$ have their usual meaning (see Figure 1) [19].

If r>1, the spectrum is right-skewed; if r<1, the spectrum is left-skewed; if r=1, the spectrum is symmetric. It is important to mention that for values r≪1, the spectrum is highly skewed to the left, and if r≫1, the spectrum is highly skewed to the right [19].

### 2.4. Curvature

In a previous article [19], we proposed the curvature parameter K as a parameter that allows us to classify different time series. It is measured around the maximum of the spectra calculated with the CHJ method because we noticed that around the maximum, the curvature changes are more pronounced [19]. At any point of a graph of a function y=f(x), the curvature is

(5) $$K=\frac{\left|\frac{d^{2}y}{dx^{2}}\right|}{{\left[1+{\left(\frac{dy}{dx}\right)}^{2}\right]}^{3/2}}$$

**Figure 2:**
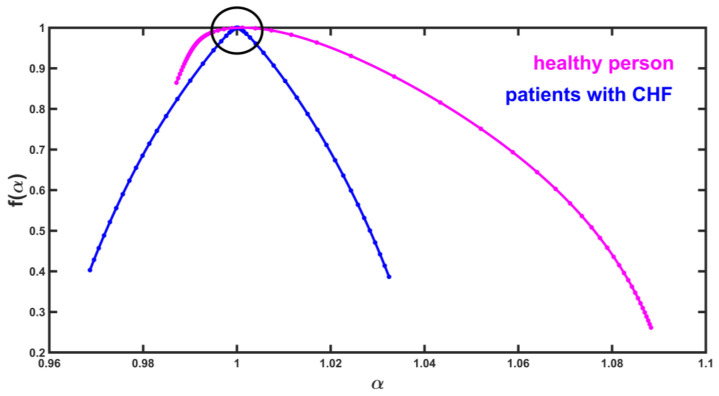
Multifractal spectra of the heart interbeat time series of a healthy subject (magenta) and a patient (blue) with congestive heart failure.

In the mentioned article, we analyzed the heartbeat time series of healthy subjects and patients with congestive heart failure (CHF), and we found that around the maximum, the multifractal spectrum has large curvature values in CHF patients and in healthy subjects, the curvature around the maximum of the spectra is small (Figure 2).

We refer the reader to reference [19] for more details about the symmetry r and curvature K parameters.

## 3. Results

### 3.1. Gutenberg-Richter Law and Cumulative Seismicity

We use the OFC model to generate magnitude synthetic earthquake series of $1\times 10^{7}$ data, with β-values from 0.01 to 0.25 for a matrix with 100×100 blocks. We used the relation [18,20,21]

(6) $${log}_{3}N=M$$

to convert the number of relaxed blocks to magnitude, where N is the number of relaxed blocks and M is the synthetic magnitude. This expression relates the area of rupture with the magnitude.

**Figure 3:**
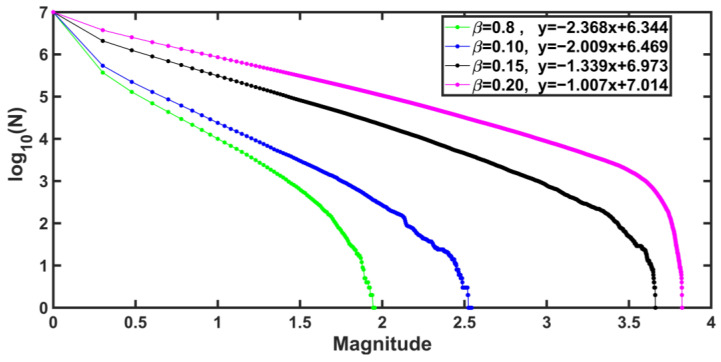
Gutenberg-Richter for values of β=0.08,0.10,0.15,0.2, respectively, as β increases b decreases, this behavior is associated with the decrease in the number of synthetic earthquakes of great magnitude as β decreases, the magnitude increases with large values of β.

In Figure 3, we show the graphics of the Gutenberg-Richter law for different values of β and observe that they have a linear trend with a negative slope. We observe that the *b*-values increase when β increases. In the Gutenberg-Richter law for β=0.2 we observe that the fit is linear with a negative slope given by b=-1.007, not far from b=-1. The value of a is 7.014.

**Figure 4:**
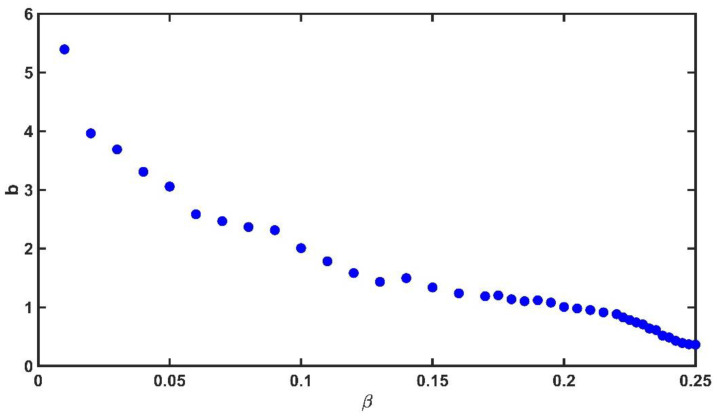
Gutenberg-Richter *b*-values as a function of β∈[0.01,0.25]. We observed that in the interval $\beta \left[0.175,0.215\right]$ the b-values are close to 1.0.

We calculated the slopes of the Gutenberg-Richter plot, and we constructed the plot of the *b*-value as a function of β (see Figure 4). We observed how the b-values decrease as β increases (see values in Table 1); this is because when taking large values of β, the magnitude of the large synthetic earthquakes increases. We observed that the b-values in the interval $\beta \epsilon \left[0.175,0.215\right]$ were close to 1.0, just as in real seismicity, the *b*-values varied around 1.0.

**Figure 5:**
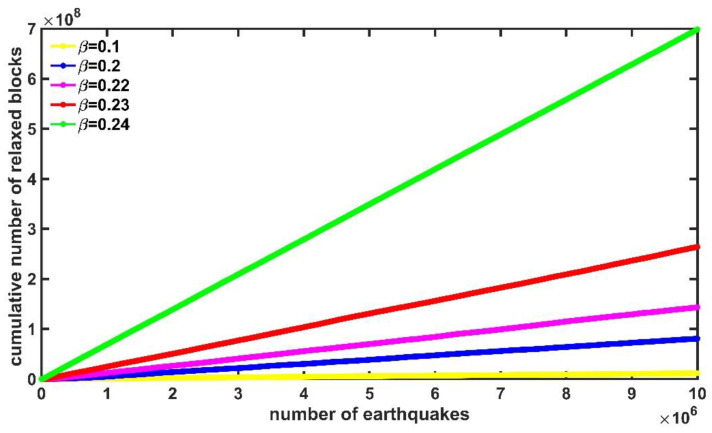
Cumulative number of relaxed blocks as a function of the number of earthquakes for β=0.1,0.2,0.22,0.23,0.24, the shown straight lines have slopes m (see Table 1).

We obtained the accumulated number of relaxed blocks as a function of the number of synthetic earthquakes for the time series with different β’s. We observed that they had a uniform linear trend in the form of a ladder which has a straight-line envelope (see Ref. [14]) for a large number of synthetic earthquakes. These straight lines have positive slopes, which means an increase in the number of relaxed blocks as the size of the series increases. Figure 5 shows the behavior of the accumulated number of relaxed blocks as a function of the number of earthquakes for values of β=0.1,0.2,0.22,0.23,0.24 of the series with $1\times 10^{7}$ data.

**Figure 6:**
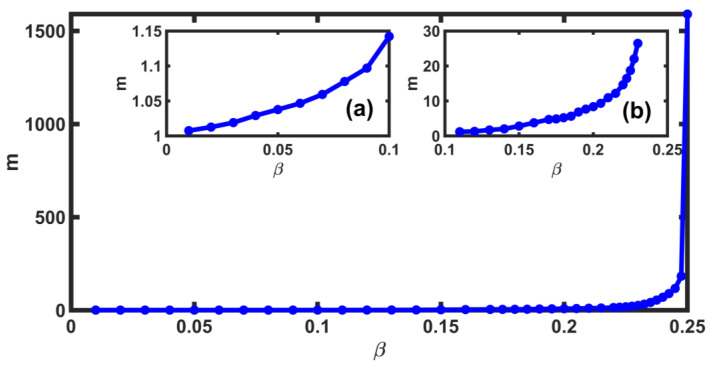
Slope *m* of the accumulated number of relaxed blocks for the different values of β∈[0.01,0.25]. The *m*-values increase as β grows. This means that the number of relaxed blocks increases for large values of β. Insets (**a**,**b**) are enlargements of the sections for β∈[0.01,0.17] and β∈[0.175,0.23].

We calculated the slope of the accumulated number of relaxed blocks as a function of the number of earthquakes for the β-values in the interval [0.01,0.25]. With these results, we constructed the graph of slopes of the accumulated number of relaxed blocks as a function of β. The value of the slope increases as it acquires large values (see Figure 6). The fact that the slope increases for the different values of β means that the number of relaxed blocks increase (see values in Table 1). Figure 6 has enlargements for [0.01,0.1] and [0.11,0.23], where it is observed that the slope values are small but different from zero.

The time series obtained from the OFC model reproduces the Gutenberg-Richter law, and it has the same behavior as cumulative synthetic seismicity of real seismicity [15].

### 3.2. Multifractal Analysis

For each of the time series with different β-values we applied the multifractal method. Once we calculated the multifractal spectra, the parameters ∆α, r and K were calculated.

**Figure 7:**
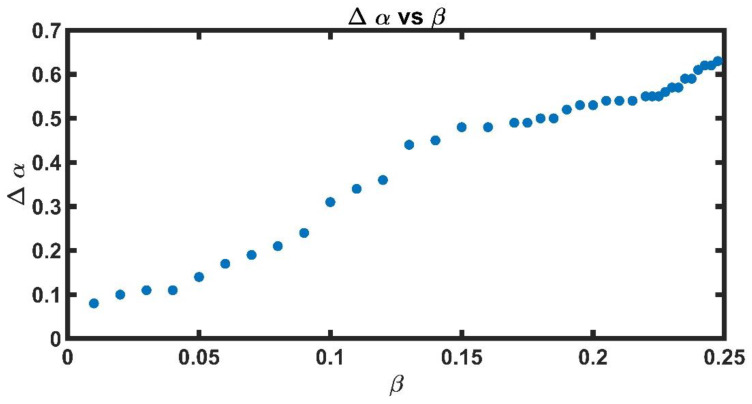
Values of ∆α as a function of β∈[0.01,0.25]. It is observed that the spectra are wider as the value of β increases.

Figure 7 shows the values of ∆α for the different values of β. It is observed that the spectra widen as the value of β grows; that is, the synthetic series becomes more complex. The values of ∆α for different β are shown in Table 2.

**Figure 8:**
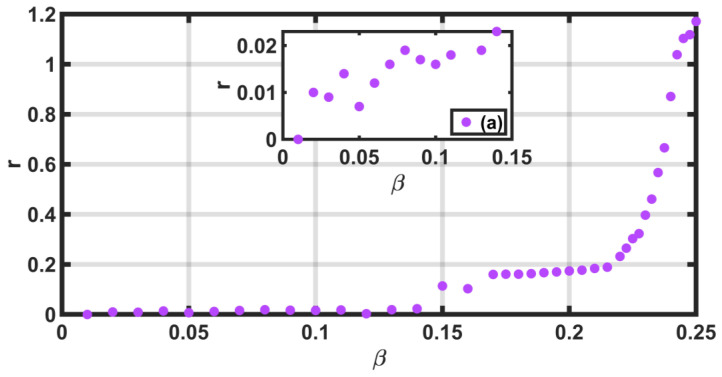
Values of r for β∈[0.01,0.25]. The Inset of the values of r as a function of β for values less than 0.15 are observed in (**a**).

We calculated r for each multifractal spectrum (see Figure 8). The values of the parameter r are represented by the vertical axis, and the horizontal axis gives the values of β. We note that the spectra corresponding to β≤0.2375 are skewed to the left. However, for values of β≥0.24, the behavior is reversed, and the spectra are skewed to the right, although they are almost symmetric, since for β=0.25, the value of r=1.171 is just slightly greater than 1.0. Inset 8 (a) is an enlargement for β≤0.15. The values of r for each value of β are in Table 2. If the spectra are skewed to the left (q>0), the most singular regions are enhanced; since the spectra of synthetic time series are mostly skewed to the left, the series have regions with many singularities, which can be interpreted as a great variability.

We observed that the *r*-values increase when β increases.

**Figure 9:**
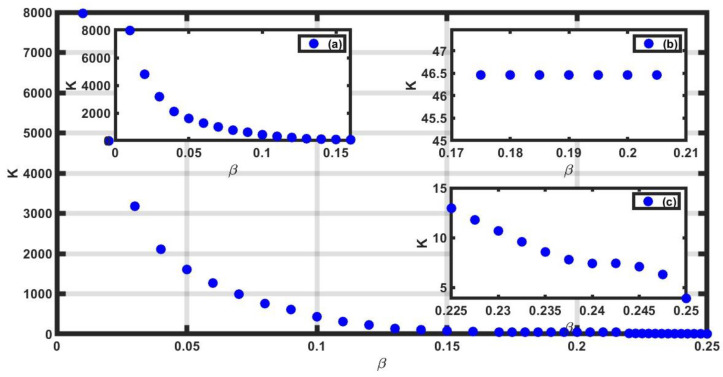
Values of K for β∈[0.01,0.25]. Inset (**a**) shows the details for β∈[0.01,0.17], (**b**) shows the details for $\beta \in \left[0.175,0.22\right],$ and (**c**) shows the details for $\beta \in \left[0.22,0.25\right]$.

Finally, we measured the curvature K around the maximum of the spectra (see Figure 9). The vertical axis is the *K*-value, and the horizontal axis gives the values of β. It is observed that the value of K for the different values of β is decreasing as it tends to 0.25. The K-values for the different β’s are shown in Table 2.

### 3.3. Studying Seismicity before and after a Large Synthetic Earthquake Using Multifractal Analysis

We performed the analysis of the synthetic earthquake time series with $1\times 10^{7}$ data for a conservation level of β=0.2 and for a matrix of 100×100 elements.

Three large synthetic earthquakes were found with magnitudes M of values of 8.01 (6640 relaxed blocks), 8.02 (6668 blocks), and 8.00 (6592 blocks). We then constructed overlapping windows around each of the synthetic earthquakes with $2^{10}$ data and an overlap of 128 data.

We applied the CHJ method to each of the windows to obtain their multifractal spectra. As in the past section, we calculated for each of them ∆α, r, and K.

**Figure 10:**
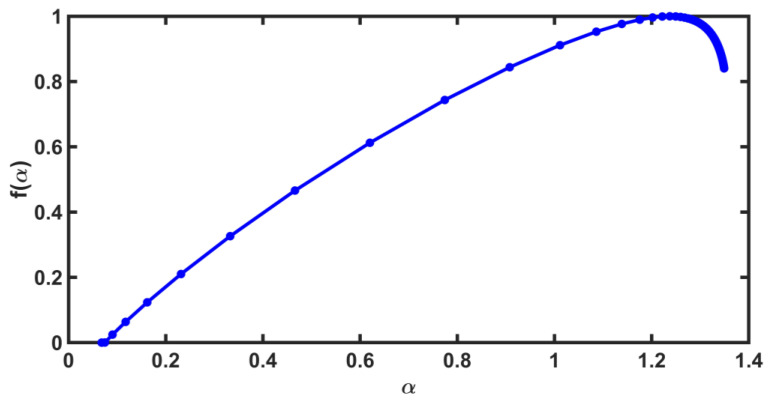
This multifractal spectrum was calculated for the window before the first large earthquake. It is skewed to the left, and it has a pronounced curvature around the maximum.

The spectra obtained with the CHJ algorithm of the synthetic series generally have the shape shown in Figure 10; that is, they are heavily skewed to the left and with a pronounced curvature around the maximum.

**Figure 11:**
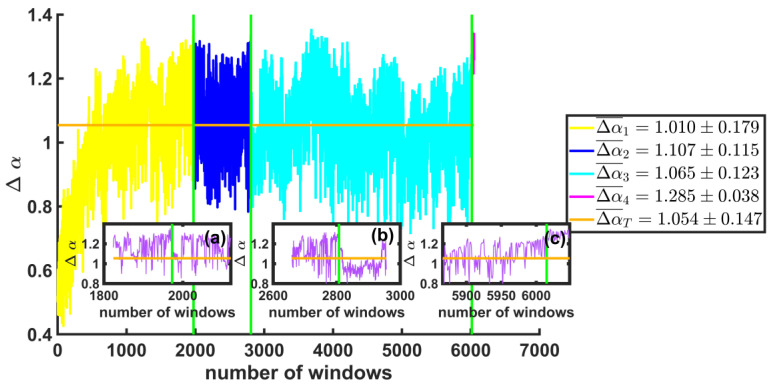
The values of ∆α for the windows before and after each of the large earthquakes are shown (vertical green lines). The orange line is the global mean of the series. Insets (**a**–**c**) are enlargements of the values of ∆α for the 150 windows after each of the first three large earthquakes. A change in the size of the widths of the spectra is noted before and after each large earthquake (M1, M2, and M3 ).

In Figure 11, we show on the right side a box with the average values of the width of the spectra, ∆α. The vertical axis shows the values of ∆α for the windows before and after each of the large earthquakes, and the horizontal axis represents the total number of windows. The orange line is the average. Insets (a), (b), and (c) are enlargements of the values of ∆α for the 150 windows after each of the first three large earthquakes (M≥8) found in the time series. We observe that the width values before the earthquake are not as wide. $\bar{{\left(∆\alpha \right)}_{1}}=1.010\pm 0.179$ (yellow line) as after $\bar{{\left(∆\alpha \right)}_{2}}=1.107\pm 0.115$ (blue line). The same behavior is seen in the three large synthetic earthquakes.

**Figure 12:**
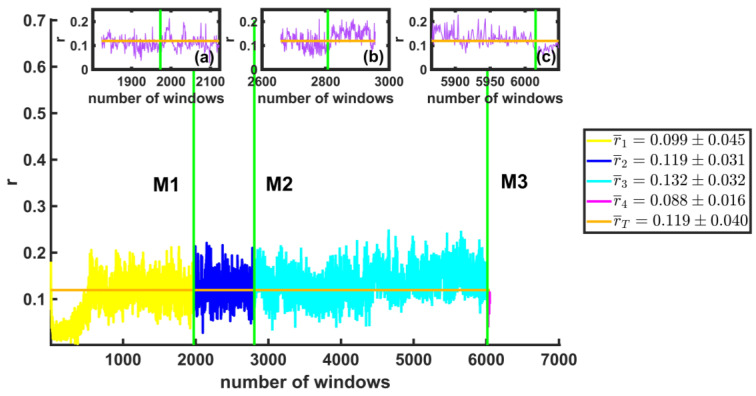
The values of r are shown for the windows before and after each of the earthquakes (vertical green lines). The orange line represents the average of *r*-values. Insets (**a**–**c**) are enlargements of the values of r for the 150 windows after each of the first three large earthquakes. It is noted that the spectra of the windows are very skewed to the left.

We calculated the symmetry parameter r of the multifractal spectra for each of the windows. In Figure 12, the average values of r are shown. The vertical axis shows the r-values for the windows before and after each of the large earthquakes, and the horizontal axis represents the total number of windows. The orange line is the average. Insets (a), (b), and (c) are enlargements of the values of r for the 150 windows after each of the first three large earthquakes found in the time series. We observed that the r-values are very small, which means that the multifractal spectra are highly skewed to the left (see orange line $r_{T}=0.119\pm 0.040$). We also note a slight change in this value between earthquakes before and after large earthquakes, as observed in insets (a), (b), and (c).

**Figure 13:**
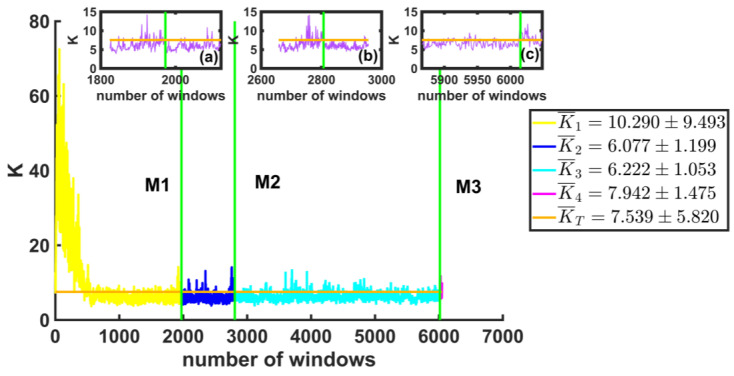
The K values for the windows before and after each of the large earthquakes are shown (vertical green lines). The orange line represents the mean of K for the series. Insets (**a**–**c**) are enlargements of the values of K for the 150 windows after the large earthquakes. The box gives the average values of K for the four intervals of windows, that is, (0,1973), (1974,2807), (2808,6015), and (6016,6048).

Finally, we calculated the curvature around the maximum of each of the windows before and after each earthquake. In the box on the right side of Figure 13, we show the average values of K. The vertical axis shows the K-values for the windows before and after each of the large earthquakes (green vertical lines), and the horizontal axis represents the total number of windows. We observed that at the beginning of the series, the windows have very large K-values of the order of 73, which means that they are highly pointed, skewed to the left, and narrow. However, as the windows grow closer to the first large earthquake, they stabilize at a curvature value of less than 10. We also observed in this case that the statistical error is very large, which shows the dispersion of the results. Like the previous parameters, in this case, we noticed a change in K-values before and after the earthquakes (see the insets (a), (b), and (c) in Figure 13).

A statistical analysis (t-Student, with a level of significance of 0.05) was made to see if the differences before and after the large-magnitude earthquakes of the parameters ∆α, r, and K were statistically significant, and it emerged that the differences are significant for r and K but not for ∆α. From the results obtained, we can infer that there is a process of preparing for the large earthquake and that the dynamics after the large earthquake are different from the previous one due to differences in the values of the parameters ∆α, r. and K for the different windows. This motivated us to apply the previous analysis to real seismicity.

### 3.4. California Seismicity

**Figure 14:**
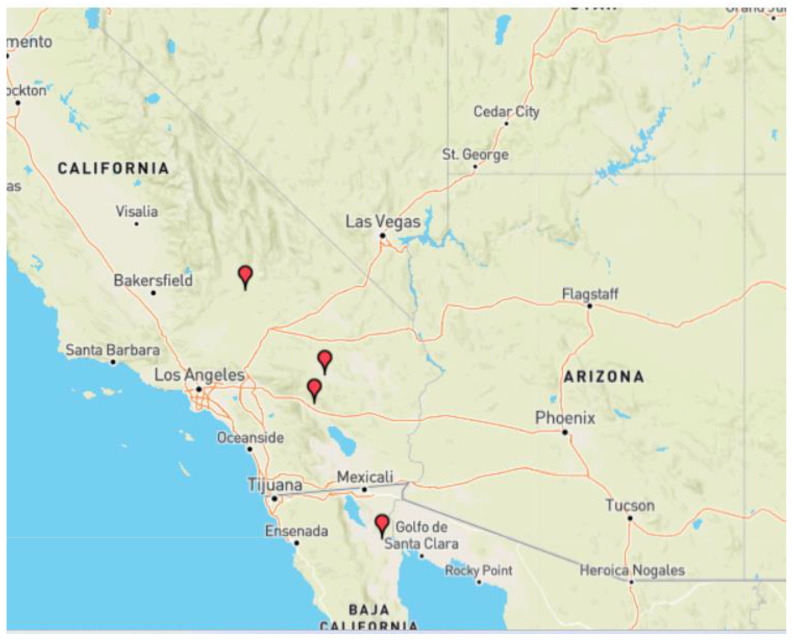
Red globes represent the location of events of magnitude greater than 7.0 in Southern California between the years 1980 to 2019. The Figure was taken from the website Google Map Display of Earthquake Catalog Search Results from SCEDC (caltech.edu).

**Figure 15:**
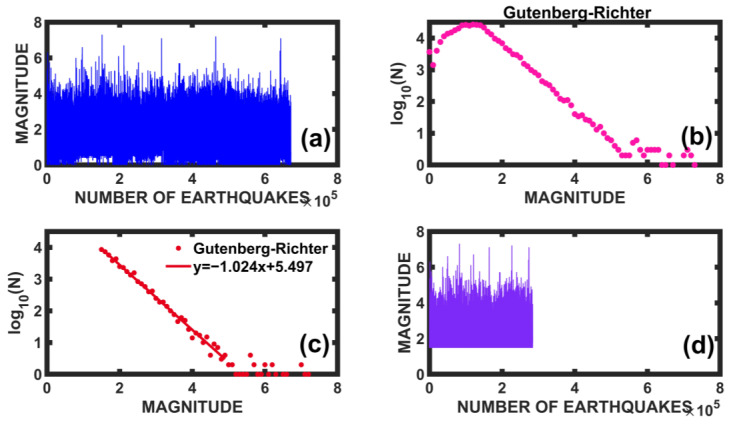
The time series of the Southern California earthquake catalog downloaded from the SCEDC website is shown in (**a**), with a total number of 671,533 events. In (**b**), the construction of the Gutenberg-Richter plot is shown with the data in (**a**). In (**c**), we observe the linear fit of the Gutenberg-Richter plot. It is noted that the catalog is complete from the magnitude value of 1.5. Finally, in (**d**), the Complete California Seismicity Catalog with 284,197 data is shown.

In this section, we study the catalog of earthquakes in Southern California for the period from 1 January 1980 to 19 August 2019, obtained from the website “The Southern California Earthquake Data Center” (SCEDC) that operates in the Seismology Laboratory at Caltech and is the primary seismological data archive for Southern California (see Figure 14). In Figure 15a, the time series that was downloaded from the website is shown, and the vertical axis represents the magnitude. For the present work, we consider large earthquakes, those with magnitude *M* greater than 7.0. The horizontal axis represents the number of earthquakes; in this case, we have 671,533 with M≥1.5.

We constructed the Gutenberg-Richter plot for this region in this time interval (see Figure 15b). As expected, in the central part of the plot, the trend is linear, with a negative slope. We proceeded to make a linear adjustment (see Figure 15c); the parameter b takes a value of −1.024, very close to -1. The value a=5.497 is a measure of the regional seismicity level. The catalog is complete from magnitude 1.5 with 284,197 earthquakes (see Figure 15d).

We looked for earthquakes with a magnitude greater than or equal to 7; in this case, four were found. The first occurred 10 km north of Yucca Valley, California, on 28 June 1992, with a magnitude of 7.3; the second occurred 16 km southwest of Ludlow, California, on 16 October 1999, with a magnitude of 7.1; the third happened 12 km in the southwest of Delta, Baja California, Mexico on 4 April 2010, with a magnitude of 7.2, and the fourth happened 18 km in the west of Searles Valley, California on 6 July 2019, with magnitude 7.1 (see Table 3 to see the detailed information of each earthquake).

Once the earthquakes of magnitude equal to or greater than 7 were located, we built windows before and after each one of them with $2^{10}$ data, and in this study, we considered overlaps of 32, 128, 512, 896, and 992. We report the results obtained with the overlap of 992 data because, in all cases, we obtain similar results.

Once we had the windows, we proceeded to make the multifractal analysis of each one of them using the CHJ algorithm, and we calculated the parameters ∆α, r, and K looking for differences before and after large earthquakes, as noted in Section 3.3.

**Figure 16:**
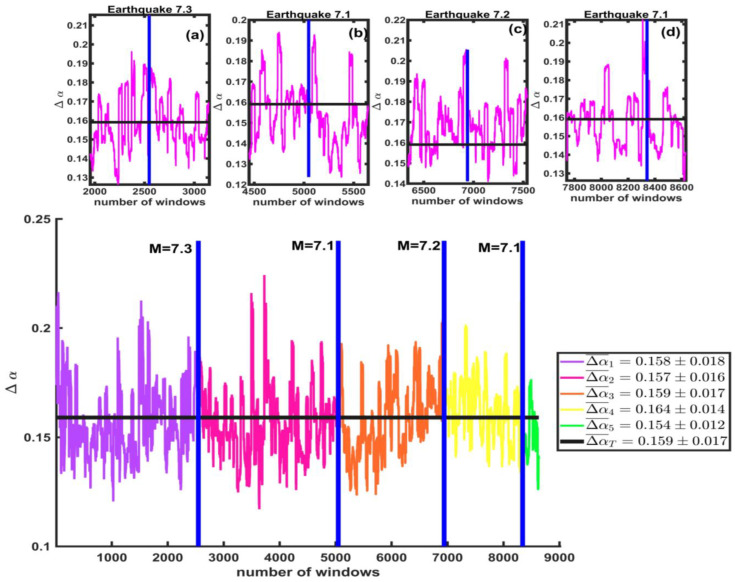
The values of ∆α for the California Seismicity catalog are shown for the windows before and after each of the large earthquakes (blue vertical line). The black line is the average of the ∆α -values. Insets (**a**–**d**) are enlargements of the ∆α values for the 600 windows before and after each of the large earthquakes. A slight change in the size of the spectrum widths is noted before and after each earthquake, but the differences are not statistically significant. In the box on the right side, we show the average values of ∆α before and after each large earthquake.

In Figure 16, the values of the spectra width are shown. The vertical axis shows the values of ∆α for the windows, and the horizontal axis represents the total number of windows. The black line is the average. Insets (a), (b), (c), and (d) are enlargements of the values of the 600 windows before and after each one of the large earthquakes, where we notice a slight change in the value of the width size before and after each earthquake.

The average values of the width before and after the earthquake are very similar in the M7.3 earthquake; the average value before is $\bar{{∆\alpha }_{1}}=0.158\pm 0.018$ (purple line), and after, is $\bar{{∆\alpha }_{2}}=0.157\pm 0.016$ (pink line). We see this same behavior again in the M7.1 earthquake since the average value before is $\bar{\Delta \alpha_{2}}=0.157\pm 0.016$ (pink line), and after, it is $\bar{\Delta \alpha_{3}}=0.159\pm 0.017$(orange line). For the M7.2 earthquake, we observe that these values are different before and after, that is, $\bar{\Delta \alpha_{3}}=0.159\pm 0.017$ (orange line) and $\bar{\Delta \alpha_{4}}=0.164\pm 0.014$ (yellow line). It happens in a similar manner for the last M7.1 earthquake, where the values are $\bar{\Delta \alpha_{4}}=0.164\pm 0.014$ (yellow line) and $\bar{\Delta \alpha_{5}}=0.154\pm 0.012$ (yellow line).

**Figure 17:**
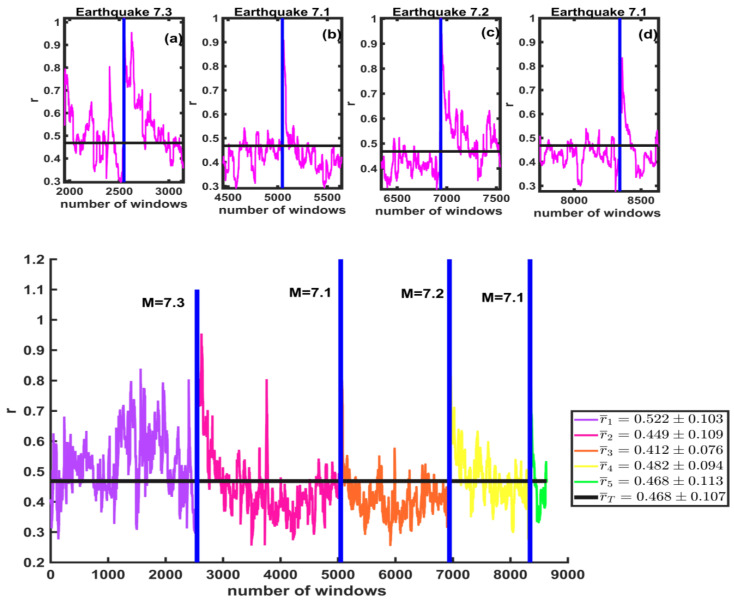
The r-values are shown for the windows for each of the large earthquakes (vertical blue lines). The black line represents the total average for the analyzed series. Insets (**a**–**d**) are enlargements of the 600 windows before and after each of the large earthquakes.

We calculate the symmetry parameter r for each of the windows of the multifractal spectra. In Figure 17, the average values of r are shown. The vertical axis shows the values of r for the windows, and the horizontal axis represents the total number of windows. The black line is the total average $\bar{r_{T}}=0.468\pm 0.107$; this means that the spectra are skewed to the left. We also find that the average values for the windows before and after the first large earthquake M7.3 are $\bar{r_{1}}=0.522\pm 0.103$ (line purple) and $\bar{r_{2}}=0.449\pm 0.109$ (pink line). For the second large earthquake, M7.1, these values are $\bar{r_{2}}=0.449\pm 0.109$ (pink line) before and $\bar{r_{3}}=0.413\pm 0.076$ (orange line) after. For the third large earthquake, M7.2, they are $\bar{r_{3}}=0.413\pm 0.076$ (line orange) before and $\bar{r_{4}}=0.482\pm 0.094$ (yellow line) after. Lastly, for the M7.1 earthquake, the average values are $\bar{r_{4}}=0.482\pm 0.094$ (yellow line) and $\bar{r_{5}}=0.468\pm 0.113$ (green line) before and after, respectively. Insets (a), (b), (c), and (d) are enlargements of the 600 windows before and after each of the large earthquakes from the studied seismicity catalog of California. A change in this value is noted before and after the large earthquakes, as seen in the insets (a), (b), (c), and (d), where the r-value increases after each large earthquake.

**Figure 18:**
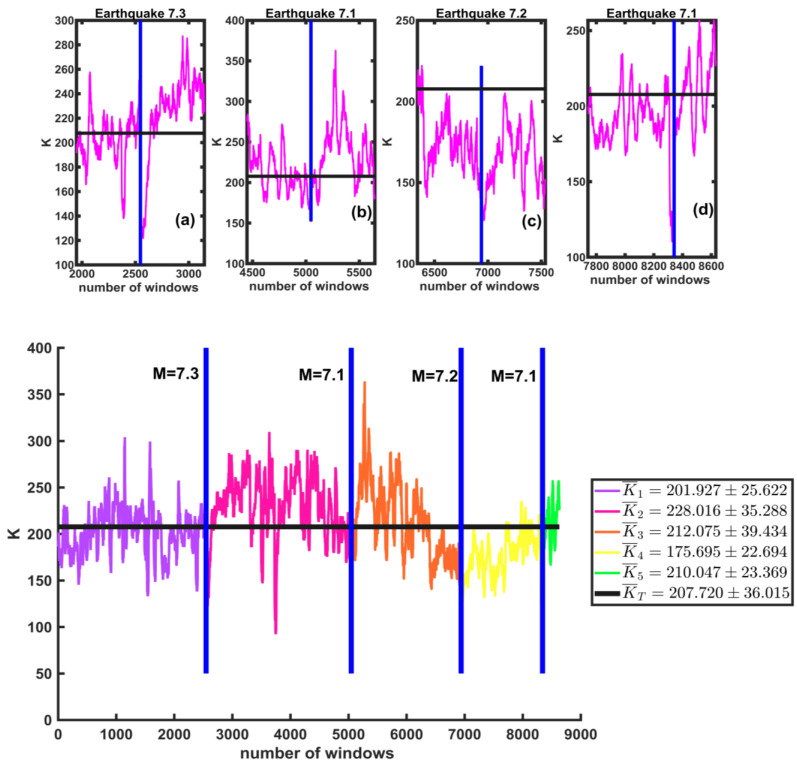
The K values for the windows before and after each of the earthquakes are shown (vertical blue lines). The black line represents the global mean of the series. Insets (**a**–**d**) are enlargements of the 600 windows before and after each of the earthquakes.

Finally, we calculated the curvature around the maximum of each of the windows in the California catalog. In Figure 18, the vertical axis shows the K values for the windows, and the horizontal axis represents the total number of windows. The black line is the total mean $\bar{K_{T}}=207.720\pm 36.015$. Insets (a), (b), (c), and (d) are magnifications of the values of the 600 windows before and after each of the four earthquakes (7.3, 7.1, 7.2, and 7.1). We observe that, unlike *r*, in this case, the *K*-value decreases immediately after the earthquake.

We also find that the average curvature value before and after the first M7.3 earthquake is $\bar{K_{1}}=201.927\pm 25.622$ (purple line) and $\bar{K_{2}}=228.016\pm 35.288$ (pink line), respectively. For the second M7.1 earthquake, the mean values before and after are $\bar{K_{2}}=228.016\pm 35.288$ (pink line) and $\bar{K_{3}}=212.075\pm 39.434$ (orange line). The average values before and after the third M7.2 earthquake are $\bar{K_{3}}=21.075\pm 39.434$(orange line) and $\bar{K_{4}}=175.695\pm 22.694$ (yellow line). Finally, for the fourth M7.1 earthquake, the mean values before and after are $\bar{K_{4}}=175.695\pm 22.694$ (yellow line) and $\bar{K_{5}}=210.047\pm 23.369$ (green line), respectively.

The parameters of the spectra ∆α, r, and K show differences between their behavior before and after the studied large earthquakes, as we observed in Section 3.3 for synthetic seismicity. In addition, it is highlighted that the spectra in both synthetic and real seismicity are skewed to the left.

It should also be mentioned that the last two parameters, r and K, provide a very characteristic change in value after large earthquakes, and it is precisely this change that brings us to the conclusion that there is a process of preparation for large earthquakes and that the dynamics after them are very different from the previous one.

## 4. Discussion

We analyzed synthetic seismicity time series with ${1\times 10}^{7}$ synthetic earthquakes obtained from the OFC model for different *β*-values. The synthetic series have a behavior very similar to real seismicity since the Gutenberg-Richter law can be qualitatively reproduced, and for β around 0.2, we obtain *b*-values in accordance with the values observed in real seismicity. In fact, several real seismicity properties have been qualitatively reproduced using this model [18].

Multifractal analysis has been used in the analysis of time series with very good results; in particular, in a previous article [19], we applied this type of analysis to heartbeat time series and introduced a conjoint analysis of such series in terms of three very important parameters: the width of the multifractal spectrum or degree of multifractality ∆α, the symmetry of the spectrum *r*, and the curvature around the maximum of the spectrum K.

We applied this type of analysis in this work because all the synthetic seismic series obtained with the OFC model are multifractal. Each of the obtained spectra was characterized with the three parameters, ∆α, r, and K. We noticed changes in these parameters for the different values of β; as β grows the spectra become wider; therefore, the time series are more complex. In addition, as β grows, the value of r increases and K decreases. Finally, most of the spectra are skewed to the left.

As these three parameters describe multifractal spectra in a satisfactory way, we then located, in a very long synthetic time series, the three synthetic earthquakes with the greatest magnitude, which with the definition we use for magnitude (Equation (6), have magnitudes M greater than 8.0. We then chose windows with overlap both before and after these three large synthetic earthquakes, and in each window, the three mentioned parameters were calculated. For all the windows before the earthquake, the average values of these parameters were calculated, and the same was done for all the windows after the earthquake. It turned out that there is a significant difference between the values of K and r before and after the earthquake, while such a difference is not significant for the case of the average value of the width of the multifractal spectrum. For statistical analysis, Student’s *t*-test was used for the difference in means with a significance level of 0.05.

This fact is interesting because it has been mentioned [22,23,24] that the OFC model does not produce aftershocks; however, it is very interesting that differences in the mentioned parameters are obtained before and after large events, and it is that after a large synthetic earthquake, many of the blocks remain with stress values very close to the threshold, so it seems logical that the epicenters of the following synthetic earthquakes would be in them.

We analyzed the catalog of earthquakes in Southern California for an approximate time interval of 40 years, and we observed that the multifractal spectra of the complete catalog and parts of it are also multifractal time series, just as in synthetic seismicity, and the spectra are skewed to the left and can also be characterized with the parameters ∆α, r, and K.

In this interval of time, we also located the events of greater magnitude, which in this case were four earthquakes with a magnitude greater than 7.0. The same procedure of defining overlapping windows was carried out before and after such large earthquakes, and we obtained behaviors like those obtained in synthetic seismicity; that is, on average, there are significant differences between the average values of K and r before and after the earthquakes, and we did not notice significant differences in the average width of the spectra. In real seismicity, due to the presence of aftershocks, this result was somehow expected, but not in synthetic seismicity.

## 5. Conclusions

We conclude that the time series of synthetic earthquake magnitudes obtained from the OFC model are multifractal, and the degree of multifractality or width of the multifractal spectrum increases as the level of conservation β increases, which physically means that the complexity of such series increases with increasing β∈[0,0.25].

This model qualitatively reproduces the Gutenberg-Richter law, and the value of the parameter *b* of that law decreases as β increases, which means that for small values of β, synthetic earthquakes of small magnitude are produced, while if β grows, large synthetic earthquakes are produced.

In previous works, we have shown that a SOC model of the OFC type is capable of qualitatively reproducing various properties of real seismicity [9,14,20,21]. In the present study, we add a new property, which consists of showing that both the series of magnitudes of real seismicity, as well as the series of magnitudes of synthetic earthquakes obtained with a SOC model exhibit a concomitant multifractal behavior corresponding to their great complexity. This fact was analyzed using the parameters ∆α, r, and K, which, as seen mainly through r and K, it was possible to identify that there are variations between their values before and after large synthetic and real earthquakes, respectively. It is very interesting to see that while for large real earthquakes, it can be accepted that there are very likely geological causes for a period of preparation for the large event, this also apparently occurs for large synthetic earthquakes. Clearly, this possible coincidence needs further investigation.

## Figures and Tables

**Table 1:** Values of the Gutenberg-Richter b -values and *m*-slopes of the cumulative number of relaxed blocks for different values of β.

β	b	m	β	b	m
0.01	2.92	1.01	0.19	0.89	6.85
0.02	2.50	1.01	0.195	0.88	7.68
0.03	2.15	1.02	0.2	0.86	8.37
0.04	2.07	1.03	0.205	0.84	9.33
0.05	1.76	1.04	0.21	0.83	10.94
0.06	1.58	1.05	0.215	0.81	12.20
0.07	1.48	1.06	0.22	0.79	14.63
0.08	1.41	1.08	0.2225	0.78	16.44
0.09	1.35	1.10	0.225	0.76	18.72
0.1	1.29	1.14	0.2275	0.74	22.05
0.11	1.23	1.21	0.23	0.72	26.47
0.12	1.14	0.09	0.2325	0.70	32.72
0.13	1.03	1.72	0.235	0.68	41.10
0.14	1.01	2.02	0.2375	0.66	54.71
0.15	0.99	2.83	0.24	0.63	69.95
0.16	0.95	3.76	0.2425	0.61	89.60
0.17	0.93	4.72	0.245	0.58	117.41
0.175	0.92	4.87	0.2475	0.57	183.19
0.18	0.91	5.22	0.25	0.56	1590.21
0.185	0.90	5.64			

**Table 2:** Values of ∆α, r, and K for different values of β.

β	∆α	r	K
0.01	0.08	0.000	771.85
0.02	0.10	0.01	4802.24
0.03	0.11	0.00	3178.01
0.04	0.11	0.01	2107.60
0.05	0.14	0.01	1604.91
0.06	0.17	0.01	1267.23
0.07	0.19	0.0	991.05
0.08	0.21	0.01	757.03
0.09	0.24	0.02	609.33
0.1	0.31	0.02	429.64
0.11	0.34	0.02	309.55
0.12	0.36	0.00	225.70
0.13	0.44	0.02	136.70
0.14	0.45	0.02	105.41
0.15	0.48	0.11	80.00
0.16	0.48	0.10	61.03
0.17	0.49	0.16	46.46
0.175	0.49	0.16	46.46
0.18	0.50	0.16	46.46
0.185	0.50	0.16	46.46
0.19	0.52	0.17	46.46
0.195	0.53	0.17	46.46
0.2	0.53	0.17	46.46
0.205	0.54	0.18	46.46
0.21	0.54	0.18	46.46
0.215	0.54	0.19	46.46
0.22	0.55	0.23	15.37
0.2225	0.55	0.27	14.219
0.225	0.55	0.30	12.957
0.2275	0.56	0.32	11.79
0.23	0.57	0.40	10.68
0.2325	0.57	0.46	9.58
0.235	0.59	0.57	8.57
0.2375	0.59	0.67	7.80
0.24	0.61	0.87	7.41
0.2425	0.62	1.04	7.42
0.245	0.62	1.10	7.09
0.2475	0.63	1.12	6.30
0.25	0.84	1.17	3.90

**Table 3:** Information on earthquakes of magnitude greater than 7.0 from the California catalog in the considered period.

Date	Hour	Magnitude	Latitude	Longitude	Depth (km)
28 June 1999	11:57:34.13	7.30	34.20	−116.44	−0.1
16 October 1999	09:46:44.46	7.10	34.60	−116.27	13.7
04 April 2010	22:40:42.36	7.20	32.29	−115.29	10.0
06 July 2019	03:19:53.04	7.10	35.77	−117.60	8.0

## Data Availability

The database can be provided by writing to the corresponding author.
